# Exploring time evolution characteristics of the collaborative mode in emergency information release of public health emergencies: A network analysis of response to COVID-19 from the central government of China

**DOI:** 10.3389/fpubh.2022.970514

**Published:** 2022-08-29

**Authors:** Jida Liu, Yuwei Song, Shi An, Changqi Dong, Chenxi Lian

**Affiliations:** ^1^School of Management, Harbin Institute of Technology, Harbin, China; ^2^Research Center for Public Safety and Emergency Management, Harbin Institute of Technology, Harbin, China

**Keywords:** public health emergency, emergency information release, time evolution, COVID-19 pandemic, social network analysis, emergency collaboration

## Abstract

Emergency information release during public health emergencies is a governance measure to slow down the spread of the epidemic and guide the public in scientific protection. Because of the uncertainty and life-cycle characteristics of public health emergencies, emergency information release represents the process of time dynamics. At present, it is an inevitable trend to establish a collaborative mechanism for emergency information release of public health emergencies to improve the release efficiency and respond to public demand. To determine time evolution characteristics of organizational collaboration in emergency information release, this study took the response to COVID-19 from the central government of China as an example and conducted research based on social network analysis. Based on information from COVID-19-related press conferences held by China's central government, the emergency information release collaborative networks (EIRCNs), and Emergency Organizations-Emergency Information Release Matters (EOs-EIRMs) 2-mode network were constructed. With the time evolution, the tightness, convergence, stability, and connectivity of EIRCNs in public health emergencies presented the process of lowering and then raising. At different stages, the core emergency organization (EO) nodes in EIRCNs continued to maintain a certain degree of activity. Their dynamic processes showed the characteristics of diversification rather than homogeneity. The time evolution of emergency information release matters (EIRMs) reflected the dynamic adjustment of the government's prevention and control measures and responded to the diversification of the public's understanding and protection needs during different stages of the COVID-19 pandemic. The study further examined the driving factors and implementation mechanism of the time evolution characteristics of the collaborative mode of emergency information release. The implementation of EIRMs at different stages had different resource requirements, which were usually achieved by introducing new EOs (Adding resource increment) or increasing the collaborative frequencies among EOs (Activating resource stock). In addition, further research prospects and feasibility interpretation were proposed.

## Introduction

The COVID-19 pandemic is the most extensive pandemic afflicting humanity in a century, which is a typical public health emergency (1). As a severe global crisis and a daunting challenge, it poses a grave threat to human life and health (2, 3). Public health emergencies are often characterized by uncertainty, sprawl, and rapid spread (4). In responding to public health emergencies, the government and relevant departments should release emergency information to the public promptly so that the public can fully understand and participate in the prevention and response work of public health emergencies (5). The emergency information release is the main channel for the government to communicate with the public and regulate public opinion and a critical window for the public to obtain authoritative information in public health emergencies (6). In practice, the Regulations on Emergency Response to Public Health Emergencies promulgated by the Chinese government explicitly propose establishing an emergency information release system. It is crucial to enhance the ability to release emergency information and guide the public to implement the protection of public health emergencies to prevent and reduce the loss of public health emergencies, safeguard public safety, and maintain social stability (7, 8).

Due to the coupling and evolution of the social environment and risk types, the emergency management of public health emergencies also presents complexity and systematicness (9, 10). Correspondingly, the issue content, coverage, and dissemination channels of emergency information release of public health emergencies have become more complex. Any single organization can no longer meet the resource needs of emergency information release. Thus, there is an urgent need for effective collaboration among emergency organizations (EOs) at different levels and fields to provide a multi-organizational guarantee, multi-source data support, and multi-resource supply for emergency information release (11, 12). Strengthening organizational collaboration is beneficial to breaking down departmental barriers in emergency information release and integrating information and data scattered in various fields. Furthermore, because of the uncertainty of public health emergencies and the variations of the virus (13), the organization mode and strategy selection of emergency information release show a dynamic process of continuous change and constant adjustment (14). Therefore, by paying attention to the time dynamic characteristics of public health emergencies, it is critical to clarify the collaborative relationship among organizations. It is also crucial to improve the efficiency of emergency information release, effectively respond to the safety needs of the public, and slow down the spread of public health emergencies.

At the present stage, to clearly explain and describe the interaction among various functional organizations in emergency management, the study of emergency management networks has been widely concerned by the academic community (15). Network analysis can be used to explain and examine the relationship among EOs (16). Therefore, this study built the EIRCNs and EOs-EIRMs 2-mode network based on information from COVID-19-related press conferences held by China's central government. By analyzing the characteristics of the structure of EIRCNs, the role and location of EO nodes, and the affiliation relationship between EOs and EIRMs in different stages of COVID-19 emergency management, the time evolution characteristics of the collaborative mode in emergency information release of public health emergencies were interpreted.

The remainder of this study is organized as follows: Section Literature review discusses and summarizes the existing studies on emergency information release and emergency management networks and puts forward the feasibility and necessity of this study. Section Methodology and data introduces the research framework and introduces the data source, the proposed analysis method, and key indicators. Section Results presents the main analysis results of this study, including the stage characteristics of the structures of EIRCNs, the attribute characteristics of EO nodes, and the affiliation relations between EOs and EIRMs. In Section Discussion, time evolution characteristics of the collaborative mode of emergency information release are summarized from a system perspective, and influencing factors of network evolution are further interpreted. Section Conclusions and recommendations summarizes the conclusions, contributions, and limitations of the study, as well as discusses the research implications.

## Literature review

### Studies on emergency information release

As its main aim is to support disaster emergency response, maintain social and public stability, and mitigate the loss of disasters, emergency information release has always been a vital issue in academic and governance practice. Meanwhile, with the rapid development of mobile communication, internet, social media, and other emerging media (17, 18), society and public demand for emergency information is further intensified. This presents new requirements and challenges for emergency information release (19). Broadening the channels, clarifying the content, improving the efficiency, and completing the mechanism of emergency information release have become essential research objectives in the academic circles.

Emergency information can be divided into data information about emergencies, emergency response measures taken by the government and relevant departments, emergency protection measures taken by the public, and public service information related to emergencies (20). The emergency information release runs through the whole life cycle of emergency management (21). This includes the release of early warning information in the stage of emergency prevention, the release of disaster information as well as prevention and control measures in the stage of emergency response and disposal, and the report of accident investigation and experience summary in the stage of emergency recovery. The credibility of information sources, the accuracy of information content, and the timeliness of information release are considered the key to evaluating the level of emergency information release. Existing studies have included the emergency information release into the system of government information disclosure and attributed the emergency information release to active disclosure rather than an application for disclosure (22). Meanwhile, it should be noted that the central government and the local government have different characteristics of emergency information release. Among them, the local government mainly includes provincial governments, municipal governments and community administration departments (23, 24). The existing studies discussed the characteristics of information disclosure through local communication in 31 capital cities in mainland China by describing and comparing the officially released content regarding local epidemic situations (25). The study noted that cities directly administered by the central government performed better in terms of timely reporting and the transparency of information disclosures. This indicates that the central government's participation is very important, and it is valuable to clarify the characteristics of the central government's emergency information release.

Research on emergency information release belongs to the interdisciplinary research content, involving journalism and communication science, management science, and security science. In the research of emergency information release strategy, existing studies have discussed the effect of implementing emergency information release relying on different release channels and media types, such as press conferences, social media, and information release platforms. It is also essential to pay attention to the difference between the subject and object of emergency information release and consider the particularity of public behavior, such as public response and public adaptability. The mechanism of emergency information release is the core foundation to support the implementation of emergency information release. In the research of emergency information release mechanisms, relevant research is usually conducted from the practical and theoretical levels. On the one hand, the characteristics and practical problems of the current mechanism are discussed and summarized around the system design and policy arrangement of emergency information release. On the other hand, the theoretical frameworks of emergency information release mechanisms are designed based on governance theory, integral government theory, and collaborative theory. There are also studies on the mechanism of emergency information release in different types of emergencies and levels of government departments.

### Studies on the emergency management network

To more specifically identify the types and scale of organizations involved in emergency management and explain the interaction relationships of EOs during emergency response, scholars have published several theoretical and case study results about the emergency management network (26, 27). A network is an organizational structure with multiple agents and polymorphic nodes. It is often used to solve problems that a single organization cannot solve independently (28). Previous studies have shown that different levels, types, and regions of emergencies all have a certain impact on the formation and scale of the emergency management network.

For the types of research objects, the research of emergency management networks can be divided and summarized according to the types of emergencies, such as earthquakes, floods, hurricanes, forest fires, explosion accidents, public health emergencies, and terrorist attacks. Du et al. (27) analyzed emergency response network of hazardous chemical accidents from network characteristics, organization functions and organization positions. Chen et al. (29) discussed the emergency management network of Wenchuan earthquake 2008, Yushu Earthquake 2010, Lushan Earthquake 2013 and Ludian Earthquake 2014. The study explains the complex adaptive process and main characteristics of emergency management network. By compared the emergency collaborative networks formed by the 2015 and 2016 Myanmar floods, Htein et al. (30) concluded that the network structures changed from military-centered to polycentric interactions. Lian et al. (10) took COVID-19 as the example to construct emergency cooperative networks of supply support and analyzed the expansion mechanism of the institutional network, the interactive network, and the social network.

For the construction methods of network construction, the academic circles primarily use texts of emergency response plans, actual case reports, and policy texts as data sources to identify types of organizations participating in emergency management. They construct interaction relationships between emergency response organizations to analyze constructing emergency management networks. By analyzing the interaction between EOs, the emergency management network can be constructed. Fan et al. (31) took government contingency plans as the research subjects, the research sample of 110 contingency plans of F District in Shanghai were collected. Accordingly, the research analyzed cross-agency collaboration based on the theory of network embeddedness. Zhang et al. (32) collected relevant data of the Ya 'an Earthquake from five data sources: situation reports, local newspapers, live news conferences, internet news, and social media so as to construct the emergency response network. Niu et al. (33) designed the interagency response network based on policy documents during the emergency response, which were searched by identifying the relevant keywords about COVID-19 in official website.

For the research design, the network structure and node attributes have always been critical analysis perspectives of the emergency management network. Among them, network structure characteristics can effectively reflect the scale of cooperation and evaluate the cooperation performance of EOs (34, 35), whereas node attributes are a vital basis for differentiating the functions of EOs (36). Nodes in the emergency management network can be divided into government departments, social organizations, and the public according to the types of EOs. The relationships among government departments at different levels and between government departments and social organizations or the public are the primary source of connected data in the emergency management network. Scholars have not only used statistical description and graphical representation to analyze the emergency management network but also extended the research perspective to explore the formation mechanism of the network. Kim et al. (37) studied the risk communication networks for the South Korean government in response to MERS based on the Exponential Random Graph Model (ERGM). Reciprocity and transitivity of network relationship, organization level and regional organization are considered to be the main factors affecting the formation of emergency network. Song et al. (38) analyzed disaster-resilient networks built in Seoul Metropolitan City based on Quadratic Assignment Procedure (QAP). The results show that the common political attributes of cities, the similarity of urban emergency capacity, and the similarity of urban environmental vulnerability are the important influencing factors of network development. Jung et al. (39) explored the dynamics structure of the interorganizational emergency management network based on the stochastic actor-based model. Reciprocity of network relationships and joint participation in large-scale pre-disaster exercises will affect the formation of networks, which supports the interdependent risk hypothesis.

In addition, based on life cycle characteristics of emergency management, the emergency management network shows certain differences in different stages, which are regarded as the dynamic characteristics of the network (40). On the one hand, existing research has obtained the dynamic evolution law of emergency management networks by sorting out the evolution mode of the network. Relevant conclusions can provide theoretical and data support for the dynamic adjustment of emergency management decisions. On the other hand, by comparing the emergency management network constructed based on emergency plans or actual cases, scholars have explored the similarities and differences between the target state and practice mode. They have provided an observable analysis path for the learning feedback mechanism of emergency management after the event. Liu et al. (26) examined the 7 March building collapse in Quanzhou City, China as the case, and discussed the dynamic evolution characteristics of the emergency collaboration network for compound disasters. Du et al. (27) exploring the time dynamics of emergency response network for hazardous chemical accidents was explored by taking the Jiangsu Xiangshui 3.21 chemical plant explosion accident as the example. Lu et al. (41) analyzed the dynamic characteristics of emergency inter-organizational communication network under public health emergency by taking the COVID-19 pandemic in Hubei Province of China as the example.

Emergency collaboration is essential to improve the performance of emergency management (42). As a vital component of emergency management, emergency information release needs organizational collaboration to support (43). The collaboration of emergency information release plays a positive role in strengthening the interaction level of EOs, realizing the integration of heterogeneous emergency resources, and improving the efficiency of emergency information release. However, the analysis and research on the collaborative mode and characteristics of emergency information release are rare. Moreover, the studies on the cooperation of EOs in public health emergencies have often focused on the issues of emergency supplies scheduling, epidemic prevention and control, and emergency rescue. There is relatively little research on the relationship among EOs in the emergency information release of public health emergencies. Therefore, this study took the response to COVID-19 from the central government of China as an example to investigate the collaboration and interaction among EOs in the emergency information release of public health emergencies based on social network analysis. It explored time evolution characteristics of the EIRCNs of public health emergencies from the dynamic perspective of the development of public health emergencies.

## Methodology and data

### Research design and framework construction

The Chinese government has adopted different modes and core contents of emergency information release at various stages of COVID-19 emergency management (44). The present study analyzed the time evolution characteristics of the collaborative mode of emergency information release from the network perspective. In terms of dividing the time interval, this study referred to the classification rules in *Fighting COVID-19 China in Action White Paper* and divided the collaboration process of emergency information release of COVID-19 into five stages (see Table 1). In Stage I, only sporadic COVID-19 cases occurred in Wuhan but did not spread to the whole country. Therefore, there were no large-scale EIRCN among EOs in Stage I. The State Council Information Office held its first press conference on January 22, 2020, in Stage II. To sum up, this study focused on the time evolution characteristics of the EIRCNs from Stage II to Stage V.

**Table 1:** Review of the Chinese government's response to COVID-19.

**Stage**	**Time interval**	**Basic characteristics**
Stage I	December 27, 2019-January 19, 2020	Swift response to the public health emergency
Stage II	January 20-February 20, 2020	Initial progress in containing the virus
Stage III	February 21-March 17, 2020	Newly confirmed domestic cases on the chinese mainland drop to single digits
Stage IV	March 18-April 28, 2020	Wuhan and Hubei – an initial victory in a critical battle
Stage V	Since April 29, 2020	Ongoing prevention and control

**Figure 1:**
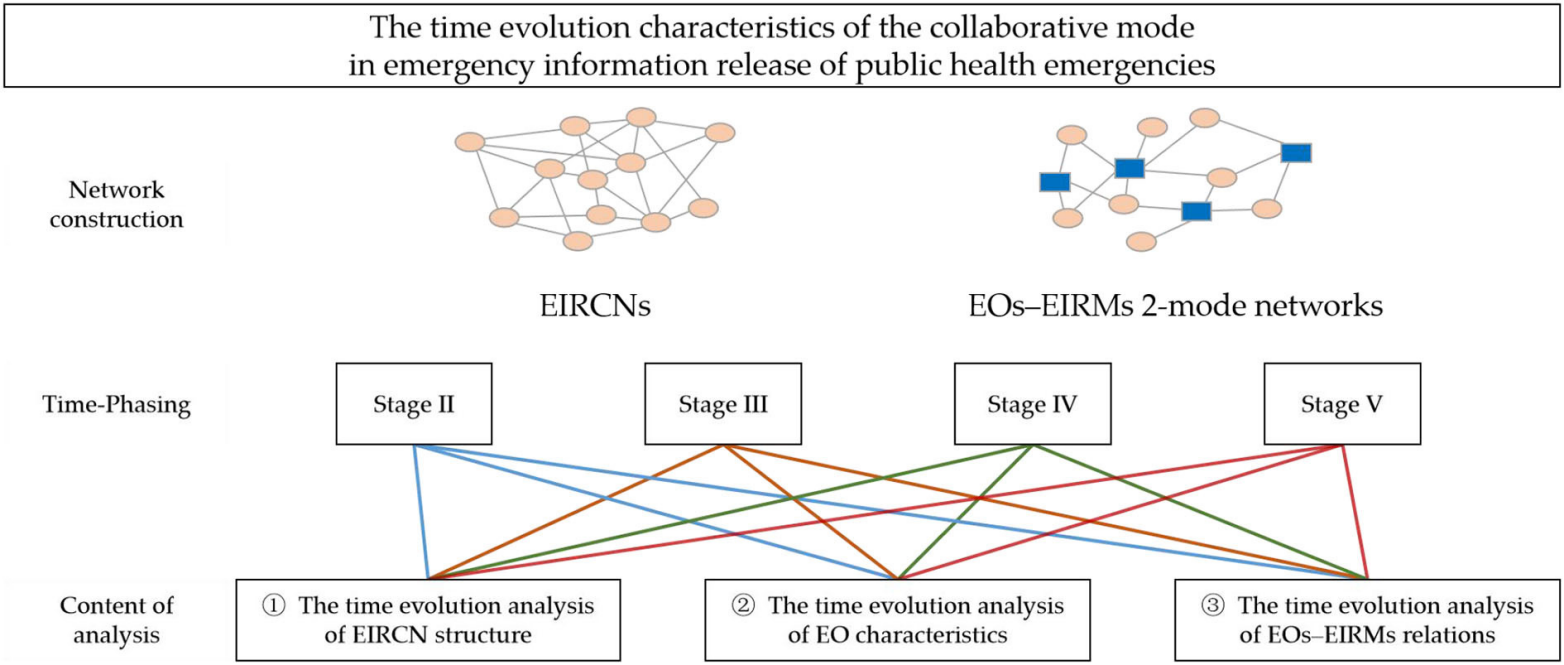
Research framework of EIRCNs.

To solve the research problems, this study proposed a time evolution of the network analysis framework (see Figure 1), which mainly included the following four steps. First, the texts of press conferences on COVID-19 prevention and control held by the State Council Information Office and the Joint Epidemic Prevention and Control Mechanism of the State Council were collected and evaluated. Thus, the relationship among EOs and the affiliation relationship between EOs and EIRMs were analyzed. Next, the EIRCNs and EOs-EIRMs 2-mode networks were constructed. Second, the EIRCNs at different stages were drawn. The time evolution characteristic of network structure was analyzed, and the dynamic development characteristic of collaborative relationships among EOs was described. Specifically, the evolution characteristics of the tightness degree among EOs, network agglomeration, and network connectivity were analyzed based on network density, network centralization, average path length, and other indicators. Third, degree centrality, betweenness centrality, closeness centrality, and eigenvector centrality were selected to analyze the position and role of EOs in EIRCNs. The comparative analysis explored the core and essential EO types from Stage II to Stage V. Fourth, because of different types of EIRMs in different epidemic prevention and control stages, the study built an EOs-EIRMs 2-mode network to analyze evolution characteristics of the EOs-EIRMs relations.

### Data collection and acquisition

This study was based on information from the press conferences related to COVID-19 held by the State Council Information Office and the Joint Epidemic Prevention and Control Mechanism of the State Council during China's response to COVID-19. The Joint Epidemic Prevention and Control Mechanism of the State Council is a multi-ministry coordination mechanism platform launched by the central government in response to the outbreak of COVID-19 in 2020. It is managed by the National Health Commission and consists of 32 departments. Under the Joint Epidemic Prevention and Control Mechanism, there are working groups on epidemic prevention and control, medical treatment, scientific research, publicity, foreign affairs, logistics support, and forward work.

On January 22, 2020, the State Council Information Office held its first press conference on COVID-19, which introduced epidemic prevention and control. To ensure the integrity and validity of research data, 201 pieces of relevant press conference information were retrieved from the website of the State Council from January 22, 2022, to March 22, 2022. Correspondingly, Stages II, III, IV, and V held 36, 37, 46, and 82 press conferences, respectively. Through analyzing the host organization of the press conferences, 107 EOs, including government departments, public institutions, and social organizations were analyzed and identified. Based on the participation and response of EOs in the press conferences, the collaborative relationships among EOs in the emergency information release were evaluated. In this study, 1,113 pairs of collaborative relationships were formed among EOs.

According to the National Public Health Emergency Plan and other relevant documents, combined with the Joint Epidemic Prevention and Control Mechanism of the State Council and the functions of the National Health Commission, 17 items of EIRMs for public health emergencies were identified, as shown in Table 2.

**Table 2:** Classification of EIRMs.

EIRM1 Epidemic data.	EIRM2 Implementation of epidemic prevention and control.	EIRM3 Popularization of emergency science.
EIRM4 Epidemic prevention and control experience.	EIRM5 Major anti-epidemic medical supplies.	EIRM6 Publicity and implementation of policies.
EIRM7 Measures for social services and security.	EIRM8 Measures for businesses returning to work.	EIRM9 Measures to maintain market order.
EIRM10 Measures for financial and fiscal support.	EIRM11 Key science and technology program.	EIRM12 Measures to secure living standards.
EIRM13 Measures to stabilize employment.	EIRM14 Prevention and control measures for key population groups.	EIRM15 Prevention and control measures for key locations.
EIRM16 Prevention and control measures for key time points.	EIRM17 Prevention and control measures for imported cases.	

### Research methods

The collaborative matrix of EOs and the affiliation matrix of EOs-EIRMs were constructed by identifying the interaction among EOs and the corresponding relationships between EOs and EIRMs in the press conference about the COVID-19 pandemic. Therefore, the EIRCNs and EOs-EIRMs 2-mode networks were drawn.

#### Network structure analysis

The network structure can express the overall overview of the collaborative mode of emergency information release. It can also describe the collaborative relationships among EOs. This study selected network density, relative network density, collaborative depth, network centralization, network cohesion, and average path length to analyze EIRCNs of different stages and condense the time evolution trajectory of EIRCNs.

(1) Network density refers to the ratio of the number of connections between nodes in the network to the maximum number of connections. The more the connections between nodes, the higher is the network density in the same network scale. When the number of links among nodes is consistent, increasing the network scale will lead to a decrease in network density. For EIRCNs, the higher the network density was, the closer were the collaborative relationships between EOs. In contrast, when the network density is lower, the connections among EOs are less and the network structure is closer.

(2) By combing the data of collaborative relationships among EOs obtained in this study, the collaborative relationships between EOs were affected by association frequency. Therefore, EIRCNs were the multi-valued network. The previous concept of network density could not consider the scale of communication and the actual frequencies of connections. The concepts of collaborative depth and relative network density were introduced to further identify the actual connection strength and tightness among EOs in the network. The collaborative depth is defined as the ratio of the actual collaborative frequencies and the number of connections among nodes. Relative network density is defined as the ratio of the actual collaborative frequencies and the maximum number of network connections. In other words, the value of relative network density is equal to the network density multiplied by the collaborative depth.

(3) Network centralization is the measure to verify whether there are core nodes in the network, which can measure the clustering trend of EIRCNs to one or more core EO nodes. In this study, degree centralization was chosen as the representative for analysis, which was equal in value to the ratio of the total of the actual difference and the total of the maximum difference of the network node degree centrality. When EIRCNs have high network centralization, the network structure generally has core-periphery characteristics. In contrast, when the network centralization of EIRCNs is low, there is no obvious core EO node, and the network structure shows balanced characteristics on the whole.

(4) Network cohesion could describe the dependence of the overall network on core nodes and the balance of network structure. When the network cohesion of EIRCN was low, there was inequality among EOs in the network. The network stability was poor and tended to the dispatching structure. Meanwhile, the power and information of the whole network were relatively concentrated. In contrast, when the network cohesion of EIRCN was high, the network had a uniform structure and was not affected by individual nodes. Resources and information in EIRCN were relatively scattered.

(5) Average path length refers to the average associated distance among the EOs in EIRCN, which can express the strength of network connectivity. When the average path length of EIRCN is higher, the communication distance among EOs in the network is longer and the cost of resource transmission is higher. In contrast, when the average path length of EIRCN is lower, the connectivity of the overall network is better. The communication among EOs are not easily affected by other factors, and the costs of forming cooperative relationship are low.

#### Node attribute analysis

Node attribute analysis was used to describe the role of EOs in EIRCNs. In this study, degree centrality, betweenness centrality, closeness centrality, and eigenvector centrality were selected to analyze EO nodes in different stages. The study intuitively and quantitatively expressed the position and measured the importance of EOs in EIRCNs in terms of numerical size and ranking. When the network scale is different, the centrality in various graphs is not comparable. Therefore, this study took normalized centrality as the analysis index.

Degree centrality refers to the number of other nodes directly connected to the EO node. In a directed network, degree centrality can be divided into in-degree and out-degree centrality. When the degree centrality of the EO node is larger, it indicates that the node is in the center of the EIRCN and has more network power.

Betweenness centrality is a measure of the extent to which the EO node is located in the middle of other pairs of nodes. Betweenness centrality can express the control of the EO node on the network resources and the interaction among nodes in EIRCN. When the betweenness centrality of the EO node is higher, it indicates that the EO is on more geodesic pairs of nodes and has mastered the transmission mode of information and resources of EIRCN.

Closeness centrality is the indicator to measure to what extent the EO node is free from the control of other nodes. Closeness centrality is the reciprocal of the average distance between the EO node and all other nodes in the EIRCN. Numerically, when the closeness centrality of the EO node is smaller, it indicates that the node is not the core point of EIRCN. When the closeness centrality of EO node is larger, the node is stronger regarding information resources, power, prestige, and influence.

Eigenvector centrality measures the importance of the EO node by comparing the centrality of adjacent nodes. When the EO node has neighboring nodes with strong influence, the eigenvector centrality of the EO node is larger. The eigenvector centrality focuses on the analysis of connection quality rather than the connection quantity of the EO node. The EO node with a larger eigenvector centrality has a higher potential value.

## Results

### The time evolution analysis of the EIRCN structure

**Figure 2:**
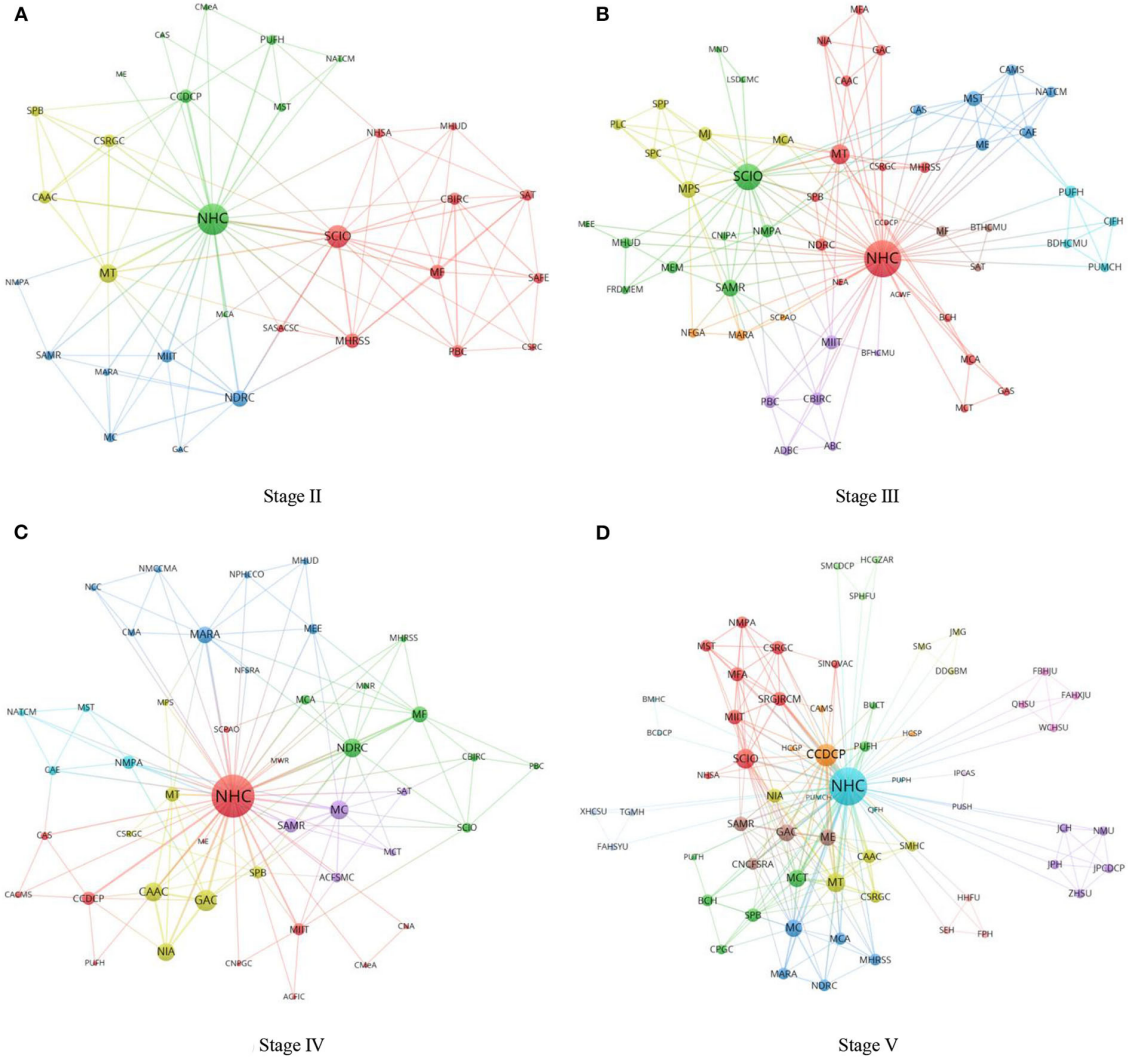
The emergency information release collaborative networks of Stages II-V. **(A)** Stage II, **(B)** Stage III, **(C)** Stage IV, and **(D)** Stage V.

To analyze time evolution characteristics of network structure, the EIRCNs of Stage II-Stage V were plotted in sequence (see Figure 2). Table 3 reports network structure indicators, including the network size, links, actual collaborative frequencies, network density, relative network density, collaborative depth, network centralization, network cohesion, and average path length.

**Table 3:** Structure characteristics of EIRCNs.

**Indicators**	**Stage II**	**Stage III**	**Stage IV**	**Stage V**
Network size (nodes)	31	55	45	63
Links	101	167	132	223
Actual collaborative frequencies	175	210	226	502
Network density (%)	21.72	11.25	13.33	11.42
Relative network density (%)	37.63	14.14	22.83	25.70
Collaborative depth	1.733	1.257	1.712	2.251
Network centralization (%)	69.43	78.65	85.94	91.49
Network cohesion	0.591	0.535	0.558	0.557
Average path length	1.890	2.013	1.919	1.886

From the perspective of basic network attributes, the EIRCNs of Stages II to V contained 31, 55, 45, and 63 EO nodes, respectively. Meanwhile, links between EO nodes in EIRCNs were 101, 167, 132, and 223 in sequence. Accordingly, the network densities of the EIRCNs of Stages II to V were 21.72%, 11.25%, 13.33%, and 11.42%, respectively. From the perspective of the actual collaborative frequencies, 175, 210, 226, and 502 pairs of collaborative relationships were formed among the EOs in Stages II to V, respectively. Correspondingly, both the relative network density and the collaborative depth tended to decline and then rise. This was because in the early stages of the COVID-19 pandemic prevention and control, EOs needed to make immediate emergency response measures to contain the development of the epidemic. Furthermore, because of the lack of familiarity with the characteristics of COVID-19, various EOs often participated in the emergency information release together, forming relatively intensive relationships. Therefore, EIRCN had the highest relative network density (37.63%) at Stage II. When entering Stage III, the number of EOs implementing emergency information release increased, and the network scale of EIRCN expanded rapidly. This reduced the relative network density and collaborative depth of EIRCN to a certain extent but also provided climbing space for developing collaboration between EOs in EIRCN. In Stages IV and V, the relative network density and collaborative depth of EIRCN rebounded as the function division of EOs became clearer and the communication between EOs became closer. The EIRCN of Stage V had the closest connection among EOs and the largest collaborative depth (2.251).

From Stage IV to V, the network centralization of the EIRCN increased gradually. Network morphology tended to have a star structure. In the early stage of COVID-19 prevention and control, the distribution of emergency information released by EOs was not clear, and the core nodes of the EIRCN were not exact. With the advance in COVID-19 prevention and control, the types and locations of core nodes in EIRCN became gradually clear, and the network as a whole had a more significant clustering trend toward core nodes. In Stage V, EIRCN had the largest network centralization, which was 91.49%. There was little difference in network cohesion at different stages, indicating that the EIRCN structure and resource allocation tended to be stable. Specifically, network cohesion decreased slightly after the transition from Stage II to III, which was related to the increase in the types and number of EOs. However, over time, the network cohesion of the EIRCN returned to a more balanced state. Contrary to the trend of relative network density and collaborative depth, the average path length of EIRCN showed the evolution path of ascending and then descending. In Stage III, the average path length of EIRCN was the largest (2.013). This indicated that with more EOs, the communication distance and the coordination costs in EIRCN increased. Furthermore, the collaborative efficiency of EOs recovered significantly in Stages IV and V.

To sum up, with the increasing tasks for COVID-19 prevention and control, the need for EIRCN for emergency resources also deepened. The network size and the collaborative frequencies of EIRCNs showed an increasing trend. Correspondingly, EIRCN had lower relative network density, collaborative depth, network cohesion, and higher average path length at Stage III. However, with the time evolution, the division of functions was gradually clarified, and the collaborative relationships were deepened. As a result, the network tightness, convergence, stability, and connectivity of EIRCNs gradually enhanced.

### The time evolution analysis of EO characteristics

Figure 2 shows that the number, type, and location of EOs in EIRCNs vary at different stages. Therefore, to further identify key EOs involved in emergency information release, degree centrality, betweenness centrality, closeness centrality, and eigenvector centrality of EOs in EIRCNs at different stages were determined in this part. The time evolution characteristics of EO nodes could be analyzed from various aspects of the network.

Table 4 shows the top 10 EO nodes ranked by normalized degree centrality in EIRCNs at different stages. The EO node has a higher degree of centrality, indicating that the node has more direct contact with other nodes and has more active information transmission channels. In Stages II to V, NHC is the EO node ranked 1 by degree centrality because the NHC is responsible for health care and epidemic prevention and control in China. Moreover, NHC is the leading department of the Joint Epidemic Prevention and Control Mechanism of The State Council and undertook the convening of several press conferences. In addition, from Stages II to V, NHC and MT ranked in the top 10 EO nodes of degree centrality.

**Table 4:** Top 10 EOs with the normalized degree centrality in Stages II–V.

**Emergency** ** organizations**	**Stage II**		**Stage III**		**Stage IV**		**Stage V**	
	**Values**	**Rank**	**Values**	**Rank**	**Values**	**Rank**	**Values**	**Rank**
NHC	30.476	1	27.407	1	29.83	1	9.032	1
SCIO	16.667	2	11.111	2	-	-	1.29	8
MT	11.905	3	7.407	3	3.977	9	2.742	3
NDRC	9.524	4	-	-	6.534	5	-	-
MF	7.619	5	-	-	4.83	8	-	-
MHRSS	7.619	6	-	-	-	-	-	-
CCDCP	6.667	7	-	-	-	-	4.274	2
MIIT	6.19	8	4.074	8	-	-	-	-
CSRGC	6.19	9	-	-	-	-	0.887	10
CAAC	6.19	10	-	-	6.818	3	0.887	10
MST	-	-	5.556	4	-	-	-	-
SAMR	-	-	5.185	5	3.977	10	-	-
MPS	-	-	4.815	6	-	-	-	-
CBIRC	-	-	4.444	7	-	-	-	-
CAE	-	-	4.074	9	-	-	-	-
NMPA	-	-	3.704	10	-	-	-	-
PBC	-	-	3.704	10	-	-	-	-
GAC	-	-	-	-	8.239	2	1.452	7
MC	-	-	-	-	6.818	4	1.29	8
NIA	-	-	-	-	5.966	6	-	-
MARA	-	-	-	-	5.114	7	-	-
MCT	-	-	-	-	-	-	1.895	4
SRGJRCM	-	-	-	-	-	-	1.653	5
ME	-	-	-	-	-	-	1.613	6

In Stage II, EO nodes with a high degree of centrality mainly included epidemic prevention and control departments (NHC, CCDCP), news and publicity department (SCIO), transportation departments (MT, CSRGC, CAAC), and policy support departments (NDRC, MF, MHRSS, MIIT). In the face of the urgency and uncertainty of the COVID-19 pandemic, the priority of the emergency tasks in the early stage of epidemic prevention and control was stability. Consequently, the types of core EO nodes in EIRCN were relatively concentrated. In Stage III, NHC, SCIO, and MT were still the top 3 EO nodes with degree centrality. Meanwhile, market management and financial service departments (SAMR, CBIRC, PBC), science technology and medical material departments (MST, MIIT, CAE, NMPA), and public safety department (MPS) became the core organizations in this stage because of the needs of businesses returning to work, vaccine research and development, and social order maintenance. In Stage IV, the epidemic situation in China was under control. In contrast, the pandemic abroad spread rapidly, and, accordingly, the customs, immigration management departments (GAC, NIA), and civil aviation transportation departments (CAAC, MT) had a high degree of centrality. The Chinese government promoted the resumption of work and production to improve the speed and expand the scope. The functional departments such as commerce, finance, and development (MC, MF, and NDRC) were at the core of the network. In Stage V, the prevention and control of key time points and critical locations became a vital issue of concern to the government. Therefore, the tourism departments (MCT), education departments (ME), and transportation departments (MT, CSRGC, CAAC) were closer to the core of the network. SRGJRCM conducted multiple reports on COVID-19 vaccine research and public vaccination, which had a high degree of centrality.

Among EIRCNs at different stages, the top 10 EO nodes with normalized betweenness centrality are shown in Table 5. When the betweenness centrality of EO is higher, it indicates that the EO node can control the interaction and resource transfer between other non-adjacent nodes. EO nodes that were on the edge of EIRCNs did not have control over network resources and other nodes. From Stages II to V, NHC, SCIO, and MT were among the top 10 EO nodes with betweenness centrality.

**Table 5:** Top 10 EOs with the normalized betweenness centrality in Stages II–V.

**Emergency** ** organizations**	**Stage II**		**Stage III**		**Stage IV**		**Stage V**	
	**Values**	**Rank**	**Values**	**Rank**	**Values**	**Rank**	**Values**	**Rank**
NHC	57.608	1	68.153	1	75.147	1	80.598	1
SCIO	15.731	2	20.504	2	0.915	8	1.424	3
MT	2.808	3	3.589	3	0.696	10	0.885	5
NDRC	2.682	4	-	-	2.751	3	-	-
MHRSS	2.533	5	-	-	-	-	-	-
MF	2.266	6	-	-	3.794	2	-	-
NHSA	2.092	7	-	-	-	-	-	-
PBC	2.026	8	-	-	-	-	-	-
CBIRC	2.026	9	-	-	1.76	6	-	-
CCDCP	1.51	10	-	-	-	-	4.01	2
MPS	-	-	2.761	4	-	-	-	-
MJ	-	-	1.748	5	-	-	-	-
SAMR	-	-	1.639	6	0.723	9	-	-
MST	-	-	0.926	7	-	-	-	-
MHUD	-	-	0.792	8	-	-	-	-
NMPA	-	-	0.719	9	-	-	-	-
MEM	-	-	0.652	10	-	-	-	-
MARA	-	-	-	-	2.731	4	-	-
MC	-	-	-	-	2.708	5	0.915	4
MIIT	-	-	-	-	1.114	7	0.206	10
MCT	-	-	-	-	-	-	0.766	6
ME	-	-	-	-	-	-	0.544	7
GAC	-	-	-	-	-	-	0.396	8
SRGJRCM	-	-	-	-	-	-	0.241	9

In Stage II, NHC, SCIO, and MT were the top 3 EO nodes with betweenness centrality, indicating that they had more power and control f more resources in EIRCN. Moreover, the types of EOs with high betweenness centrality were mainly livelihood guarantee departments (NDRC, MHRSS, NHSA) and financial support departments (MF, PBC, CBIRC). This indicated that the implementation of various emergency tasks in the early stage of epidemic prevention and control could be separated from social and financial resources. In Stage III, NHC, SCIO, and MT still had the highest betweenness centrality, which has a similar trend with degree centrality analysis. During this stage, as the governments needed to take various measures to support the resumption of work, production, and industry, it had great demand and dependence on social order and market order. Therefore, security and legal protection departments (MPS, MJ, MEM) and marketing and industry management departments (SAMR, MHUD, NMPA) occupied more geodesic pairs of nodes in the network. In Stage IV, the spread of the epidemic in China was basically blocked. Consolidating the effectiveness of epidemic prevention and control and orderly recovery of economic and social development became the main theme of emergency information release during this stage. Therefore, in addition to finance (MF, CBIRC) and development (NDRC) departments, MARA, MC, and MIIT also provided corresponding governance resources for industrial production, employment security, and rural epidemic prevention and control. This provided the mediating effect for communication and coordination between EOs in EIRCN. In Stage V, the national epidemic prevention and control entered Ongoing Prevention and Control. The epidemic data, the introduction of experience, and emergency science popularization were vital contents of emergency information release. Therefore, NHC, CCDCP, and SCIO had a high betweenness centrality. Furthermore, departments in charge of key prevention and control locations, such as transportation, tourism, education, and customs, had more resources on the network. This also indicated that the emergency information release in this stage needed more support from heterogeneous EO and diversified governance resources.

Table 6 lists the top 10 EO nodes with normalized closeness centrality in each EIRCN at different stages. The EO nodes with higher closeness centrality have a higher possibility of establishing direct or internal connections with other nodes quickly. Moreover, the EO node does not need to obtain information and resources from other nodes. Compared with other centrality indicators, the closeness centrality of the EO node is more likely to be the same. Therefore, the rankings of EOs in different stages shown in Table 6 present the parallel phenomenon. From Stages II to V, NHC and MIIT are the top 10 EO nodes with closeness centrality.

**Table 6:** Top 10 EOs with the normalized closeness centrality in Stages II–V.

**Emergency** ** organizations**	**Stage II**		**Stage III**		**Stage IV**		**Stage V**	
	**Values**	**Rank**	**Values**	**Rank**	**Values**	**Rank**	**Values**	**Rank**
NHC	88.235	1	88.525	1	95.652	1	98.413	1
SCIO	69.767	2	65.854	2	-	-	58.491	3
MT	61.224	3	58.065	3	-	-	57.944	4
NDRC	60	4	-	-	58.667	2	-	-
MF	58.824	5	-	-	57.143	5	-	-
MHRSS	58.824	6	-	-	-	-	-	-
CCDCP	57.692	7	-	-	-	-	62	2
MIIT	56.604	8	54	7	54.321	6	54.867	9
CBIRC	56.604	8	53.465	9	-	-	-	-
PBC	56.604	8	53.465	10	-	-	-	-
NHSA	56.604	8	-	-	-	-	-	-
MPS	-	-	55.67	4	-	-	-	-
SAMR	-	-	55.67	4	54.321	6	54.867	9
MST	-	-	54.545	6	-	-	-	-
MJ	-	-	54	7	-	-	-	-
NMPA	-	-	53.465	10	-	-	-	-
ME	-	-	53.465	10	-	-	55.856	8
MC	-	-	-	-	58.667	2	56.881	5
MARA	-	-	-	-	57.895	4	-	-
GAC	-	-	-	-	54.321	6	56.364	7
MEE	-	-	-	-	54.321	6	-	-
SPB	-	-	-	-	54.321	6	-	-
MCT	-	-	-	-	-	-	56.881	5

In Stage II, the top 10 EO nodes with closeness centrality and betweenness centrality were identical in type. However, the top 10 EO nodes with closeness centrality were different in order. This indicated that EO nodes on the geodesic pair of nodes had a higher likelihood of establishing connections with other nodes. More precisely, EO nodes with more network resources in EIRCN are often not controlled by other nodes. Compared with Stage II, in Stage III and Stage IV EIRCNs, six new types of EO nodes were added to the top 10 EO sets, respectively. EIRCN of Stage III corresponded to expanding the EO nodes, including MPS, SAMR, MST, MJ, NMPA, and ME. In the EIRCN of Stage IV, MC, MARA, GAC, MEE, SPB, and SAMR were the newly added top 10 EO nodes. This showed that with the abundance of EIRMs and the expansion of EIRCN, the EO sets that were not easily controlled by other nodes in the network vary greatly. In Stage V, different from the previous two stages, the top 10 EO nodes did not increase significantly. However, it showed similar integration characteristics with previous core EOs, including NHC, SCIO, MT, CCDCP, MIIT, SAMR, ME, MC, GAC, and MCT. NHC, SCIO, MT, CCDCP, and MIIT are the core EOs formed by EIRCN of Stage II. SAMR and ME were the key EOs formed by EIRCN of Stage III. MC and GAC were highly influential EOs formed by EIRCN of Stage IV. This indicated that with the advance in epidemic prevention and control and the development of time, types of EO nodes with high reputation and core influence tend to be stable with the network layout of EIRCN.

The top 10 EO nodes of normalized eigenvector centrality in EIRCNs at different stages are identified, as shown in Table 7. The EO node with higher eigenvector centrality indicates that the node forms a collaborative relationship with more influential nodes and has more high-power neighbor nodes. It also reflects that the EO node is more likely to become the core of EIRCN. From Stages II to V, NHC and MT are the top 10 EO nodes with eigenvector centrality.

**Table 7:** Top 10 EOs with the normalized eigenvector centrality in Stages II–V.

**Emergency** ** organizations**	**Stage II**		**Stage III**		**Stage IV**		**Stage V**	
	**Values**	**Rank**	**Values**	**Rank**	**Values**	**Rank**	**Values**	**Rank**
NHC	118.361	1	124.62	1	122.718	1	112.291	1
SCIO	36.908	2	16.82	4	-	-	9.986	9
MT	32.063	3	28.026	2	13.792	9	28.201	3
CCDCP	29.012	4	-	-	17.294	8	65.786	2
NDRC	25.387	5	-	-	19.887	6	-	-
MIIT	19.181	6	12.727	9	-	-	-	-
MHRSS	17.445	7	-	-	-	-	-	-
CAAC	15.865	8	-	-	26.851	3	8.57	10
CSRGC	15.865	9	-	-	-	-	-	-
MF	11.886	10	-	-	13.003	10	-	-
MST	-	-	21.071	3	-	-	-	-
CAE	-	-	15.557	5	-	-	-	-
MCA	-	-	13.646	6	-	-	-	-
MARA	-	-	13.374	7	19.875	7	-	-
SAMR	-	-	12.842	8	-	-	-	-
CBIRC	-	-	12.347	10	-	-	-	-
GAC	-	-	-	-	30.768	2	15.971	7
NIA	-	-	-	-	23.535	4	-	-
MC	-	-	-	-	20.315	5	10.145	8
SRGJRCM	-	-	-	-	-	-	21.924	4
MCT	-	-	-	-	-	-	20.425	5
ME	-	-	-	-	-	-	19.28	6

Comparing Tables 7 and 4, we conclude that the types of EO nodes with high eigenvector centrality and high degree centrality in EIRCNs at different stages are highly consistent, with only one node difference (MPS). This indicated that in EIRCNs, the core EO nodes often had both highly influential collaborative partners and high-quality collaborative relationships. Because of different priorities and needs of COVID-19 prevention and control in different periods, the EOs with high-quality collaboration showed the trend of diversification. Moreover, we found that functional EO nodes and industry executive EO nodes in EIRCN tended to have higher eigenvector centrality. This means that the above EOs not only provided governance resources for EIRCNs but also established collaborative relationships with other high-power EO nodes to achieve resource reciprocity.

### The time evolution analysis of EOs–EIRMs relations

**Figure 3:**
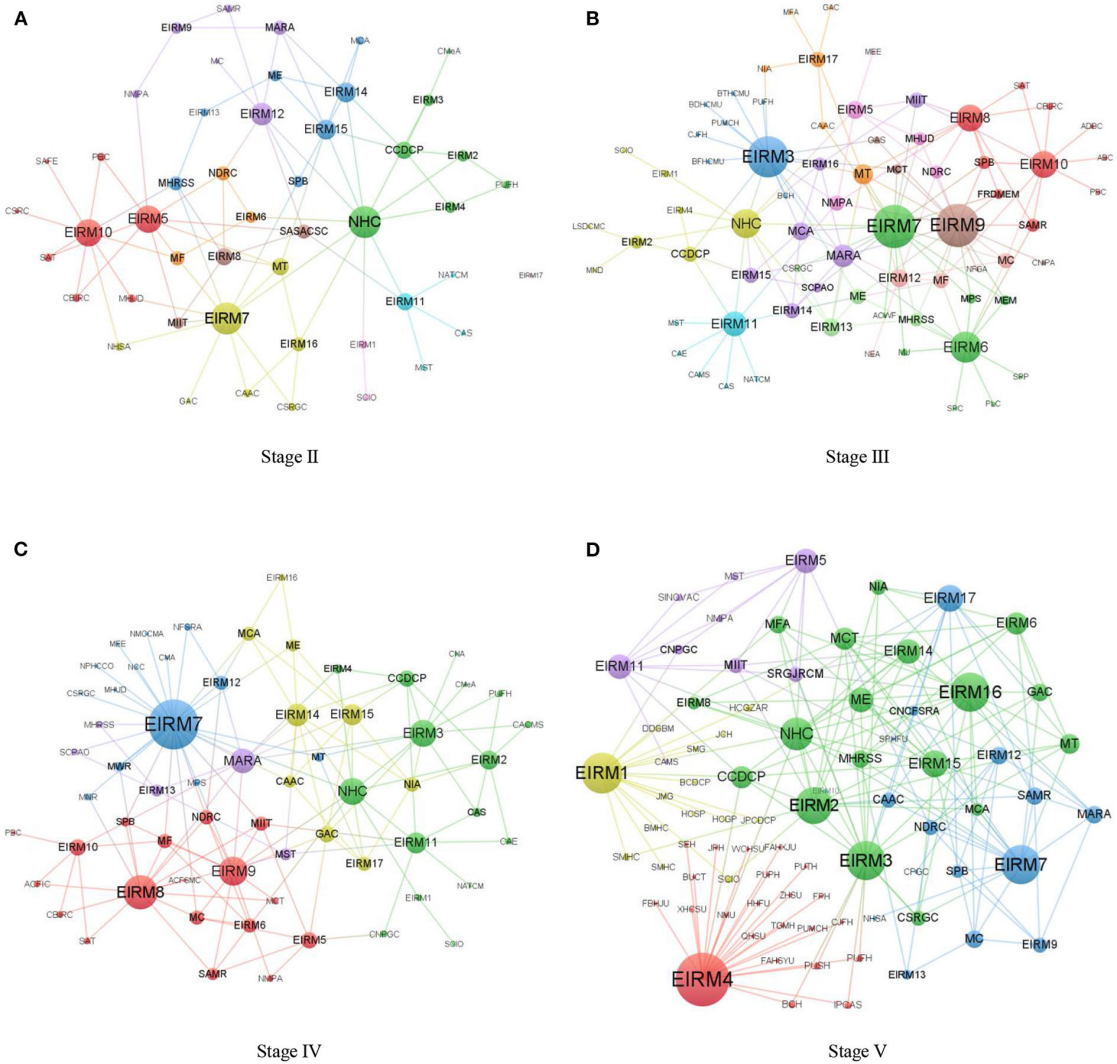
EOs-EIRMs 2-mode networks of Stages II-V. **(A)** Stage II, **(B)** Stage III, **(C)** Stage IV, and **(D)** Stage V.

To analyze differences of EIRMs at different stages and study time evolution characteristics of affiliation relations, the EOs-EIRMs 2-mode networks of Stage II-Stage V were drawn (see Figure 3). In addition, it was necessary to identify and analyze the core EIRM nodes in the network to discuss the critical focus and key issues of the EOs in the emergency information release at different stages. Therefore, the study obtained the normalized degree centrality of EIRMs from Stages II to V, as shown in Table 8.

**Table 8:** The normalized degree centrality of EIRMs in Stages II–V.

**EIRMs**	**Stage II**	**Stage III**	**Stage IV**	**Stage V**
EIRM1	0.065 (15)	0.036 (16)	0.044 (16)	0.270 (2)
EIRM2	0.097 (10)	0.073 (14)	0.156 (8)	0.238 (6)
EIRM3	0.097 (10)	0.309 (3)	0.244 (4)	0.254 (3)
EIRM4	0.097 (10)	0.036 (16)	0.067 (15)	0.381 (1)
EIRM5	0.29 (2)	0.109 (8)	0.133 (9)	0.143 (9)
EIRM6	0.097 (10)	0.218 (4)	0.111 (11)	0.127 (12)
EIRM7	0.355 (1)	0.327 (1)	0.533 (1)	0.254 (3)
EIRM8	0.161 (7)	0.182 (5)	0.333 (2)	0.063 (15)
EIRM9	0.097 (10)	0.327 (1)	0.267 (3)	0.079 (14)
EIRM10	0.29 (2)	0.182 (6)	0.133 (9)	-
EIRM11	0.161 (7)	0.164 (7)	0.178 (6)	0.143 (9)
EIRM12	0.226 (4)	0.109 (8)	0.111 (11)	0.095 (13)
EIRM13	0.065 (15)	0.109 (8)	0.089 (14)	0.048 (16)
EIRM14	0.194 (5)	0.091 (11)	0.200 (5)	0.143 (9)
EIRM15	0.194 (5)	0.091 (11)	0.178 (6)	0.159 (7)
EIRM16	0.129 (9)	0.073 (14)	0.044 (16)	0.254 (3)
EIRM17	-	0.091 (11)	0.111 (11)	0.159 (7)

Numbers in parentheses represent the rank of the EIRMs.

Figure 3 shows that in EOs–EIRMs 2-mode networks from Stages II to V, logarithms of affiliation relations between EOs and EIRMs are 177, 233, 246, and 431 respectively, indicating the trend of gradual increase. Furthermore, the network densities of EOs–EIRMs 2-mode networks from Stages II to V are 33.59%, 24.92%, 32.16%, and 40.24%, respectively. This indicates that the tightness of EOs–EIRMs 2-mode networks have similar time evolution characteristics to EIRCNs, which is the evolutionary path of decline followed by recovery. Table 8 shows that EIRMs involved and covered by EOs–EIRMs 2-mode networks at different stages are different. Stages II and V involved 16 ITEMS of emergency information release, while Stages III and IV included all 17 items of EIRMs. Among them, Stage II does not involve the issue of prevention and control measures for imported cases, and measures for financial and fiscal support are not the focus of emergency information release in Stage V.

Moreover, the same EIRM was issued by different types and numbers of EOs at different stages. With the development of COVID-19 prevention and control, emergency information release had different core issues and EIRMs. In Stage II, the contents of emergency information release mainly included diversified safeguard measures, production of emergency and medical supplies, and epidemic prevention and control of key population groups. Measures for social services and security, including transport services and daily supplies, were the most important, effectively addressing and ensuring people's livelihood during the COVID-19 outbreak. At this stage, the government introduced several financial support, tax relief, and other policies. EIRM7, EIRM10, EIRM5, EIRM12, and EIRM14 (EIRM15 tied) are the top 5 EIRMs with a degree centrality of Stage II.

In Stage III, EIRM7 is still the core topic of emergency information release. Specifically, the top 5 EIRMs are EIRM7, EIRM9, EIRM3, EIRM6, and EIRM8. EIRM17 appeared for the first time in EOs–EIRMs 2-mode networks, indicating that COVID-19 became a global pandemic. At this stage, the Chinese government issued several policies on epidemic prevention and control while ensuring economic and social development. Policy publicity and implementation was the key link of emergency information release during this period. Meanwhile, the EOs held press conferences on maintaining market order, resuming work and production, and strengthening employment security. During this period, the heterogeneity of EOs increased. This was related to emergency information release focusing on the contents of emergency science popularization.

After the transition from Stage III to IV, the core EIRMs in EOs–EIRMs 2-mode networks were EIRM7, EIRM8, EIRM9, EIRM3, and EIRM14. During this period, as the spread of COVID-19 was effectively controlled in China, the central government shifted the focus of COVID-19 prevention and control to key places, units, and groups, such as rural areas schools, and the aged. The Chinese government determined the strategy of preventing input from outside and rebounding from inside and held several press conferences focusing on the theme of prevention and control measures for imported cases. Meanwhile, the EOs repeatedly briefed the public on the progress of research and development of COVID-19 vaccines. Moreover, the Chinese government made active arrangements for international medical cooperation, export of medical supplies, and international cargo transportation and showed substantial care and concern for Chinese citizens abroad.

In Stage V, cases in all parts of China were under control, and the emergency response level was successively lowered. This indicated that COVID-19 prevention and control entered a phase of Ongoing Prevention and Control. The core focus of the emergency information release shifted to EIRM4, EIRM1, EIRM7, EIRM3, and EIRM16. During this period, the Joint Epidemic Prevention and Control Mechanism of The State Council invited EOs of different levels, types, and fields to share their experience and knowledge of epidemic prevention and control. On the eve of every holiday and other key time points, the epidemic prevention and control work was arranged around transportation, tourism, and other issues. During the period of Ongoing Prevention and Control, scientific research departments, medical management departments, and pharmaceutical companies regularly introduced COVID-19 vaccination to the society. In addition, in the face of the COVID-19 global pandemic, customs departments, immigration departments, civil aviation departments, and logistics departments focused on risk prevention and cold chain supervision of imported goods.

## Discussion

The above results describe the network structure of EIRCNs, the attributes of EOs, and the affiliation characteristics of EOs-EIRMs in different stages of COVID-19 prevention and control. From a system perspective, it is necessary to further discuss the time evolution characteristics of EIRCNs and EOs–EIRMs 2-mode networks.

(1) The network structure of EIRCNs was time evolution because of the differences in network size and the number of collaborative relationships between EO nodes in different stages. First, the relative network density and collaborative depth of EIRCNs showed the trend of decreasing first and then rising, which was formed by the expansion of the EIRCN's network scale and the gradual tightening of the collaborative relationships. Second, the type and location of core nodes in EIRCN were gradually observable because of the clear division of functions of EOs in the EIRMs. Therefore, the network centralization of EIRCNs showed the process of increasing over time. Third, there was little difference in EIRCN network cohesion at different stages. In comparison, Stage III had the lowest network cohesion. Fourth, the average path length of EIRCNs followed the evolution path of increasing first and then decreasing because the network expansion led to an increase in coordination and communication distance, which raised the collaborative costs. Furthermore, network connectivity recovered with the continuous improvement of identity among EOs. From Stage II to III, the relative network density, collaborative depth, and network cohesion decreased, and the average path length increased. With the advance in epidemic prevention and control, the network tightness, convergence, stability, and connectivity of EIRCN were gradually enhanced after Stage III.

(2) Different stages of emergency information release of public health emergencies had different governance resource requirements. Therefore, the types and locations of EOs in EIRCN evolved over time. On the one hand, with the deepening of the understanding of COVID-19 prevention and control, many industry and functional support departments joined. Meanwhile, different from the trend of gradual withdrawal of core organizations in the emergency management network, the core EOs in EIRCN continued to maintain a certain degree of activity and played a key role in all stages of epidemic prevention and control. Although the spread of COVID-19 was under overall control, it still showed the trend of sporadic outbreaks. Therefore, the core departments needed to undertake certain emergency management responsibilities and emergency information release at different times. On the other hand, because of the differences between emergency information release and emergency disposal, emergency rescue, and emergency response, core EOs in EIRCN did not show a homogenization trend compared with previous studies. This is because the emergency information in a certain field or direction can only be published by a single EO rather than by multiple EOs in the same field. Therefore, in the EIRCN of public health emergencies, the core EO nodes showed a diversified evolution trend of diversification.

(3) As the key issues and focus of emergency information release were closely related to the focus of epidemic prevention and control, the types of core EO and key EIRM nodes in the “Eos-EIRMs” 2-mode network at different stages evolved dynamically. In Stage II, the emergency information release mainly focused on epidemic prevention and control, social security, a guarantee of medical supplies, and key population groups prevention. Among them, social security included transportation, financial, and livelihood support. The guarantee of medical supplies included drug research and the production and transportation of medical supplies. In Stage III, with the initial control of the spread of the epidemic, the measures for businesses returning to work based on maintaining social stability were the vital issue concerned by emergency information release. It included the measures related to maintaining market order and stability, industrial chain recovery, industry support, employment of key groups, and fiscal and tax support. In Stage IV, concentrating on possible routes of transmission of COVID-19 was the focus of emergency management. Correspondingly, the emergency information release focused on prevention and control measures for key locations, units, and population groups. Meanwhile, because of the global spread of COVID-19, focusing on preventing and controlling imported cases also became the core content of emergency information release. In Stage V, with the ongoing prevention and control in China, the emergency information release focused on holidays, school opening, travel, transportation, and other issues. The development and vaccination of COVID-19 vaccines, imported cases, imported cold chain, and other conditions also received corresponding attention. To sum up, the dynamic adjustment of emergency information release reflected not only the government's prevention and control measures but also an intuitive response to the public's safety and protection needs.

**Figure 4:**
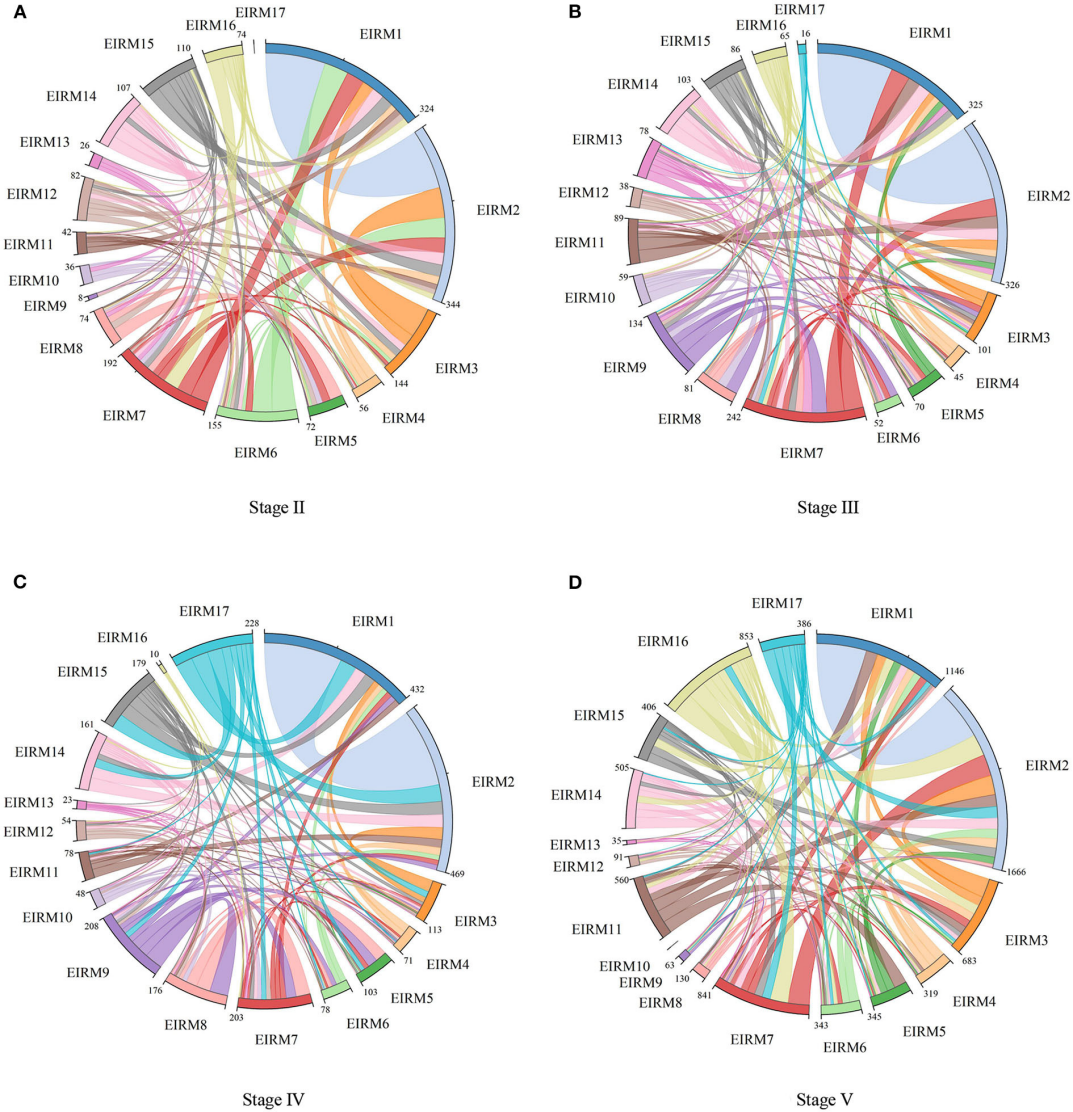
The chord diagrams of resources sharing relationship EIRMs in Stage II-V. **(A)** Stage II, **(B)** Stage III, **(C)** Stage IV, and **(D)** Stage V.

We obtained the time dynamic law of EIRCNs and EOs–EIRMs 2-mode networks. We now further discuss the factors that influence network evolution in different stages. In the emergency information release, all EOs needed to pay related network resources to realize the dissemination of EIRMs. By analyzing the interaction between different EIRM nodes, resource sharing in emergency information release can be concluded from the resource and information flow perspectives. Based on the affiliation data of EOs–EIRMs 2-mode networks, the minimums method was adopted to transform 2-mode networks to obtain the resource flow between EIRM nodes. On this basis, the chord diagrams of the resource sharing relationship of EIRMs in Stages II to V were drawn (see Figure 4). The wider the band between each pair of EIRMs, the more the emergency resources they share.

Figure 4 shows that from Stages II to V, the total amount of shared resources between EIRM nodes gradually increases. The time evolution characteristics of the total amount of network resources are related to the times of press conferences in different periods and the number of EOs participating in the emergency information release. In Stage II, EIRM2, EIRM1, EIRM7, EIRM6, and EIRM3 need to be supported by more shareable resources. By Stage III, EIRM2, EIRM1, and EIRM7 still have the highest resource sharing requirements, and EIRM9 and EIRM14 also receive more network resources. In Stage IV, in addition to EIRM2, EIRM1, EIRM9, and EIRM7, EIRM17 needs to form more resource interaction and information flow with other EIRMs and master more network resources. In Stage V of COVID-19 prevention and control, EIRM2, EIRM1, EIRM16, EIRM7, and EIRM3 are the top 5 EIRMs for controlling network resources.

Comparing Stages II and III, we find that although Stage III had many EOs, the resource sharing amount of EIRMs nodes is similar. This indicated that the high collaborative frequencies and the adequacy of interaction between EOs are also crucial ways to meet the resource requirements of EIRMs. In conclusion, different types of EIRMs at different stages and the different resource requirements of EIRMs for EOs promoted network evolution to a certain extent. Similarly, the driving forces for the time evolution of EIRCNs were similar, characterized by introducing new EOs or increasing the collaborative frequencies. Among them, introducing new EOs could improve the resource increment of EIRCNs, and the increase of the collaborative frequencies could tap the resource stock of EIRCNs.

## Conclusions and recommendations

Emergency information release during public health emergencies is a vital part of emergency management, which involves every stage of the emergency management life cycle. As the responsible public health emergency management subject, the government connected various types of EOs in emergency information release to integrate the emergency resources of different fields and attributes. The contents of emergency information were different in different stages of public health emergencies. Therefore, various EOs also formed differentiated collaborative relationships around different emergency information issues. EIRCNs and EOs–EIRMs 2-mode networks were constructed in this study by taking the response to COVID-19 from China's central government as an example. We aimed to systematically and clearly describe the coordination and interaction between EOs in emergency information release of public health emergencies. The network structure of EIRCNs and the interaction among Eos, the attributes of Eos in EIRCNs, and the relations among affiliation nodes in Eos–EIRMs 2-mode networks at different stages were analyzed. Moreover, the time evolution characteristics of the EIRCNs in public health emergencies were summarized. The research concludes the following:

(1) Comparing EIRCNs at different stages, we conclude that from Stage II to III, relative network density, collaborative depth, and network cohesion increased. In Stages IV and V, the network tightness, convergence, stability, and connectivity of EIRCNs are gradually enhanced.

(2) With the advance in COVID-19 prevention and control, the network scale of EIRCN expanded, and the types of core organizations in the network have changed constantly because of the expanding demand for emergency resources. Over time, the identified core EOs was expected to continue to maintain a certain degree of activity in the next stage rather than withdraw from the network. Moreover, the time evolution of the core EO nodes in EIRCNs showed the trend of diversification rather than homogeneity.

(3) The key issues and focus of emergency information release were closely related to the focus of epidemic prevention and control. The types of EIRM nodes in the network and their association with core EO nodes dynamically evolved. The time evolution of EIRMs in public health emergencies reflected the dynamic adjustment of the government's epidemic prevention and control measures. This also responded to the diversification of the public's understanding and protection against COVID-19 at different stages.

(4) From the time evolution characteristics of the total amount of shared resources between EIRM nodes, the driving force of the time evolution of EIRCNs was the introduction of new EOs or the increase of the collaborative frequencies between EOs. The introduction of new EOs could improve the resource increment of EIRCNs, and the increased collaborative frequencies among EOs could activate the resource stock of EIRCNs.

This study presents the following implications from two aspects: improving the collaborative mechanism of emergency information release and dynamically adjusting the key issues of emergency information release.

(1) Improve and optimize the collaborative mechanism of emergency information release from two aspects of the overall structure and local arrangement. Regarding the overall structure, institutional arrangements such as the compilation of emergency plans, function allocation, and coordination mechanism should be strengthened to improve the standardization and feasibility of organizational collaboration in emergency information release. The emergency information release policy should be updated and improved promptly. Regarding local arrangements, heterogeneous EOs should be introduced to provide diversified governance resources according to the development law of public health emergencies and the characteristics of emergency management needs. The organization foundation and trust foundation formed in emergency information release should be fully explored to avoid or reduce the repeated allocation of resources.

(2) Key topics of emergency information release should match epidemic prevention, control practices, and public safety needs. In the emergency information release of public health emergencies, EIRMs showed the time evolution feature, which revealed a high correlation with epidemic prevention and control practices. The timely and accurate release of data on public health emergencies and emergency response measures can help improve the authority and credibility of the governments and relevant departments. Furthermore, in the face of public health emergencies, the public's cognition demand for safety gradually expands. It is essential for relevant departments to timely interpret the concerning issues of public concern, such as emergency protective measures and public service information related to public health emergencies.

### Limitations

This study analyzed the collaborative mode in emergency information release from a network perspective, which is an attempt in the cross-research field of emergency management and government information release. It clarified the difference and time evolution characteristics of the organizations and contents of emergency information release in different emergency stages. This study is the first to combine the emergency information release with the network analysis method, which enriches the research on the emergency management networks. However, this study is not without limitations. The research data were mainly based on the response of the Chinese central government to the COVID-19 epidemic. There are significant differences between the central government and the local government in the authority and mode of emergency information release. Therefore, it is necessary for future research to focus on the emergency information release of the local government and clarify the difference. In addition, because of the ongoing COVID-19 pandemic, the characteristics of emergency information release in Stage V still have some extensibility. Furthermore, the collaborative mechanism of emergency information release of multiple subjects such as the governments, experts and scholars, and opinion leaders of social platforms could be used as the main content of the next research to enrich the theoretical connotation of emergency collaboration continuously.

## Data availability statement

The original contributions presented in the study are included in the article/supplementary material, further inquiries can be directed to the corresponding authors.

## Author contributions

All authors listed have made a substantial, direct, and intellectual contribution to the work and approved it for publication.

## Funding

This study was supported by the National Natural Science Foundation of China (72174044).

## Conflict of interest

The authors declare that the research was conducted in the absence of any commercial or financial relationships that could be construed as a potential conflict of interest.

## Publisher's note

All claims expressed in this article are solely those of the authors and do not necessarily represent those of their affiliated organizations, or those of the publisher, the editors and the reviewers. Any product that may be evaluated in this article, or claim that may be made by its manufacturer, is not guaranteed or endorsed by the publisher.
